# Cisplatin in breast cancer treatment in BRCA1 carriers

**DOI:** 10.1186/1897-4287-10-S4-A17

**Published:** 2012-12-10

**Authors:** Jacek Gronwald, Tomasz Byrski, Jan Lubinski, Steven A Narod

**Affiliations:** 1 International Hereditary Cancer Centre, Department of Genetics and Pathology, Pomeranian Medical University, Szczecin, Poland; 2 Women’s College Research Institute, Toronto, ON, Canada

## 

Experimental data suggest that BRCA1 related breast cancer may have increased sensitivity to platinum-based chemotherapy, but clinical data are limited. Herein we summarize our clinical observations on treatment with cicplatinum of BRCA1 mutation carriers affected with breast cancer.

## A) Neoadjuvant therapy

Eighty women with breast cancer and a BRCA1 mutation with stage I, II, and III breast cancer between December 2006 and February 2012 were entered into this study. Patients were treated with cisplatin 75 mg/m2 intravenously every three weeks for four cycles. After chemotherapy, patients underwent surgery and were assessed for pathologic response in both the breast and axillary lymph nodes. Pathologic complete response was observed in 63% of women. Conclusions: Platinum-based chemotherapy is effective in a high proportion of patients with BRCA1-associated breast cancers. Clinical trials are warranted to determine the optimum treatment for this subgroup of breast cancer patients.

## B) Treatment of metastatic breast cancer

Between July 2007 and January 2009, in a phase II, open-label study, 20 patients with metastatic breast cancer who carried a mutation in BRCA1 were treated with cisplatin 75 mg/m2 intravenously every 3 weeks as part of a 21-day cycle for 6 cycles. Restaging studies to assess response were performed after cycles 2 and 6, and every three months thereafter. Overall response rate was 80%; nine patients experienced a complete clinical response (45%) and seven experienced a partial response (35%). Overall survival was 80% at one year, 60% at two years and 25% at three years. Four of the 20 patients are alive four years after initiating treatment. The median time to progression was 12 months. The median survival from the start of cisplatinum treatment was 30 months. Cisplatin-related adverse events, including nausea (50%), anemia (5%) and neutropenia (35%) were mostly mild to moderate in severity.

## Conclusions

This phase II study demonstrates that cisplatin chemotherapy has high activity in women with a BRCA1 mutation and metastatic breast cancer and is generally well tolerated.

